# Dataset for authentication and authorization using physical layer properties in indoor environment

**DOI:** 10.1016/j.dib.2024.110589

**Published:** 2024-06-04

**Authors:** Kazi Istiaque Ahmed, Mohammad Tahir, Sian Lun Lau, Mohamed Hadi Habaebi, Abdul Ahad, Ivan Miguel Pires

**Affiliations:** a Department of Computing and Information Systems, Sunway University, Petaling Jaya, 47500 Selangor, Malaysia; b Department of Computing, University of Turku, FI-20014 Turun Yliopisto, Finland; c Centre of Research Impact and Outcome, Chitkara University Institute of Engineering and Technology, Chitkara University, Punjab 140401, India; d School of Engineering and Technology Sunway University No 5, Jalan Universiti, Bandar Sunway 47500 Selangor Darul Ehsan, Malaysia; e IoT & Wireless Communication Protocols Lab, Department of Electrical and Computer Engineering, International Islamic University Malaysia, Jalan Gombak, 53100 Selangor, Malaysia; f School of Software, Northwestern Polytechnical University, Xian, Shaanxi, PR China; g Department of Electronics and Communication Engineering, Istanbul Technical University, Turkey; h Instituto de Telecomunicações, Escola Superior de Tecnologia e Gestão de Águeda, Universidade de Aveiro, Águeda, Portugal

**Keywords:** RSSI, LQI, Authentication, Authorization, Physical layer, Machine learning, Security, Internet of things.

## Abstract

The proliferation landscape of the Internet of Things (IoT) has accentuated the critical role of Authentication and Authorization (AA) mechanisms in securing interconnected devices. There is a lack of relevant datasets that can aid in building appropriate machine learning enabled security solutions focusing on authentication and authorization using physical layer characteristics. In this context, our research presents a novel dataset derived from real-world scenarios, utilizing Zigbee Zolertia Z1 nodes to capture physical layer properties in indoor environments. The dataset encompasses crucial parameters such as Received Signal Strength Indicator (RSSI), Link Quality Indicator (LQI), Device Internal Temperature, Device Battery Level, and more, providing a comprehensive foundation for advancing Machine learning enabled AA in IoT ecosystems.

Specifications Table

**Table:** 

Subject	*Computer Engineering*
Specific subject area	*Wireless Sensor Short-Range Communication Security*
Data format	Raw, Analyzed, Filtered
Type of data	Tables, Images, Graphs, Figure
Data collection	A series of data collection experiments were undertaken methodically to get valuable insights into the radio properties of IEEE 802.15.4 device-to-device (D2D) communication. The data is collected from stationary nodes using Zigbee Zolertia Z1 with 802.15.4 low-power short-range radios. The datasets are obtained from multiple range and antenna orientation tests. The data collected includes received signal strength, link quality indicators, device internal temperature, and battery level. Moreover, device internal acceleration (X-Axis, Y-Axis, Z-Axis), Channel Check Rate, Radio Channel, and Transmission (Tx) power (in dBm) are also included. The data is presented in a reusable format for authentication and authorization security purposes.
Data source location	The experiments were conducted in the IoT & Wireless Communication Protocols Lab at the ECE/KoE/IIUM (3.252705087816474, 101.73060238904714). The location was free from obstructions and wireless activity to minimize any potential external sources of interference and disturbance that could affect the accuracy of the results.
Data accessibility	Repository Name: Dataset for Authentication and Authorization using Physical Layer Properties in Indoor Environment DOI:10.5281/zenodo.10706416 Direct URL to the data: https://zenodo.org/records/10706416

## 1 Value of the Data

• Network engineers and IoT security analysts need the data to understand the characteristics of ZigBee network at 2.4 GHz band used for many wireless indoor security applications such as protecting boot sequence, management of keys, data protection, secure sessions on communication establishments, reliable hardware/software patches, monitoring and auditing.
• These data provide valuable evidence about the impacts of the environment on the tracesets parameter of the ZigBee network at the 2.4 GHz band.
• This dataset is an input for designing and developing the ZigBee security system model that is not limited to only MAC address but also considers various physical layer features such as different angles, distances, antenna orientation, acceleration, etc.
• This dataset can serve as a standard against which future research can be carried out for several purposes. By examining RSSI, LQI, internal device temperature, and internal environmental acceleration measurements in various settings, researchers may enhance the current level of AA techniques for indoor and industrial environments. This advancement ultimately has the potential to improve a broad range of IoT security applications.
• Furthermore, the data allows for the evaluation of network security effectiveness, signal integrity, and dependability in susceptible indoor settings, creating more robust and effective secure IoT communication solutions.

## 2 Background

The creation of this dataset is motivated by the essential requirement to develop a reliable method of verifying and granting access based on physical-layer characteristics in indoor environments with no obstructions and no movement. Comprehending the physical layer characteristics of IoT devices is essential for improving network performance, increasing dependability, and extending the battery life of devices. A popular communication network that offers coverage with minimal implementation cost and power consumption is Zigbee [1], a low-power wide area networking protocol developed on top of the IEEE 802.15.4 is used to build the dataset. The dataset emphasizes important physical layer characteristics of ZigBee Zolertia Z1 Motes, such as Received Signal Strength Indicator (RSSI), Link Quality Indicator (LQI), device internal temperature, and device battery level. These characteristics are crucial in evaluating the general health and effectiveness of the communication link between IoT devices. Multiple studies have highlighted the significance of comprehending RSSI and LQI in wireless sensor networks since they directly impact communication dependability and network stability [2,3]. It is crucial to monitor the internal temperature and battery level of devices to detect failures, optimize energy usage, and ensure the lifespan of battery-powered IoT devices [4]. Inspired by the limited availability of publicly accessible datasets that included RSSI, LQI, Temperature, and Battery Level specifically designed for the security of indoor environments, our objective was to provide significant resources for researchers and professionals in this sector [5]. Methodologically, we utilized Zolertia Z1 motes and strategically located gateways to capture real-world scenarios, including the intricacies of changing environments and the dynamics inherent to indoor free-space settings.

## 3 Data Description

The ZigBee Zolertia Z1 motes utilize cost-effective communication technology for providing location information in indoor and industrial environments in device-to-device (D2D) wireless sensor networks (WSN). This is achieved by leveraging IEEE 802.15.4 technology, which provides up to 100 m indoors and up to 1000 m outdoors range, extended battery life and economical operation due to its utilization of license-free frequency channels. However, in indoor environments with short range, the coverage is quite promising and offers improved link quality.

The most common physical layer parameters utilized for authentication and authorization (AA) in low-powered devices are the Media Access Control (MAC) Address and Received Signal Strength Indicator (RSSI). Moreover, link quality indicators (LQI), internal temperature, and battery level can also play a crucial role in IoT AA systems. AA systems need expensive hardware such as multi-array antenna and phase detection, which makes it inefficient for low-cost essential IoT systems. Therefore, MAC, RSSI, and, based AA methods have become a well-known and cost-effective solution. However, RSSI and LQI are highly sensitive to environmental changes and interference. This may lead to inaccurate and unreliable AA accuracy and has more negative effects when we work in an indoor free space with a harsh environment [6,7]. As there is a lack of dataset availability with crucial physical layer parameters, we prepare our dataset for further authentication and authorization study of IoT devices. This dataset comprises radio parameters of Zigbee Zolertia Z1 802.15.4 D2D communications from a systematic collection of experiments, which provide some significant observations regarding the radio properties of Zigbee Zolertia Z1 802.15.4 D2D communications.

The dataset is organized into three folders to facilitate usability and allow future research endeavours. The RAW folder's file headers contain information on the Gateway node, including the MAC address, channel check rate, and radio channel. In addition, the gateway node measures several parameters such as temperature, environmental acceleration in the x, y, and z directions, battery level in millivolts, and transmission power. The end node measures and records parameters such as MAC, channel check rate, RSSI, LQI, Temperature, environmental acceleration in the x, y, and z directions, battery level in millivolts, radio channel, and transmission power. Table 1 provides a detailed overview of the various components present in the given dataset.

**Table 1:** Description of the captured parameters considered within the dataset.

SL	Parameters	Description
1	Capture ID	An ID
1	DateTime	Date and Time of Collected Request in Unix Timestamp
3	Gateway's MAC	Gateway/Edge node's Media Access Control (MAC) Address
4	Gateway's Channel Check Rate	Channel Check Rate of the Gateway (128 Hz)
5	Gateway's temperature (mC)	Gateway's temperature in millicoulombs
6	Gateway's Acceleration X-axis	Device Internal Acceleration due to the vibration of location in X-axis
7	Gateway's Acceleration Y-axis	Device Internal Acceleration due to the vibration of location in Y-axis
8	Gateway's Acceleration Z-axis	Device Internal Acceleration due to the vibration of location in Z-axis
9	Gateway's Battery level (mV)	Battery Level of the Gateway in millivolts (mV)
10	Gateway's Radio Channel	Gateway's Radio Channel for Communication
11	Gateway's Transmission Power (dBM)	The transmission power of broadcasting in decibels-milliwatt (dBM)
12	Gateway's Antenna Orientation	Antenna Orientation in fixed on degrees
13	Client's MAC	Client node's Media Access Control (MAC) Address
14	Client's Channel Check Rate	Channel Check Rate of the Gateway (128 Hz)
15	Client's RSSI (dBM)	Received Signal Strength Indicator (RSSI) in dBM
16	Client's LQI	Link Quality Indicator (LQI), which indicates the Link Quality
17	Client's Temperature (mC)	Client's Temperature in millicoulombs
18	Client's Acceleration X-axis	Device Internal Acceleration due to the vibration of location in X-axis
19	Client's Acceleration Y-axis	Device Internal Acceleration due to the vibration of location in Y-axis
20	Client's Acceleration Z-axis	Device Internal Acceleration due to the vibration of location in Z-axis
21	Client's Battery level (mV)	Battery Level of the Gateway in millivolts (mV)
22	Client's Radio Channel	Client's Radio Channel to Communicate
23	Client's Transmission Power (dBM)	The transmission power of the sender in decibels-milliwatt (dBM)
24	Client's Antenna Orientation	Antenna Orientation of the sender in degrees (90/0/180)

To elucidate the impact of the environment on the RSSI, LQI, and other metrics, we deployed three end nodes at fixed positions, precisely 1 m, 2 m, and 3 m away from the gateway node. The end nodes transmit packets with a consistent antenna orientation, allowing for an assessment of how the dynamic susceptible environment affects the parameters and accuracy of AA.

## 4 Experimental Design, Materials and Methods

### 4.1 Methodology

The data collection methodology employed in this study involves the examination of various metrics, including MAC, RSSI (Received Signal Strength Indicator), LQI (Link Quality Indicator), Temperature, Acceleration (X-Axis, Y-Axis, Z-Axis), Battery Level/Consumption, Channel Check Rate, Radio Channel, and Transmission (Tx) power (in dBm). These metrics are specifically chosen to facilitate the characterization of the radio environment within wireless sensor networks. The measurement of adequate contact time is an essential aspect of our study. This refers to the specific time window in which nodes can establish radio communication and possess sufficient bandwidth to facilitate data transfer. The method of collecting data is contingent upon the distance and orientation of the antenna, which is determined depending on the designated time frame for data collection. This approach is employed to assess the impact of environmental factors on indoor equipment.

The experiments were conducted within the confines of the Indoor Free Space Environment IoT & Wireless Communication Protocols Laboratory, located in the Department of Electrical and Computer Engineering at the International Islamic University Malaysia (IIUM), with the following setup:

(i) Studies were conducted in an environment free from barriers and radio interference inside the 802.15.4 radio band.
(ii) Four Zigbee Zolertia Z1 Motes were utilized for the experiments. One of the devices was designated as the receiver, while the remaining three devices were positioned as senders at distances of 1 m, 2 m, and 3 m, respectively.
(iii) Three different antenna orientations, namely 90°, 180°, and 0°, were selected depending on the distances of individual client nodes.

The nodes were affixed within the space in each instance, with no internal movement occurring. Nevertheless, individuals occasionally traversed the area beyond the laboratory enclosure at a pace consistent with walking. The trials were replicated by varying the placement of the transmitter and receiver nodes inside the confines of the laboratory. The decision to pursue this option is to measure the influence of the nodeʼs positions more prone to being transported.

### 4.2 Tracesets parameters

Each Traceset Row comprises the following information: Capture ID, Datetime, MAC address of the Gateway (Receiver), Gateway Channel Check Rate, and Gateway Temperature. The variables of interest in this study include gateway acceleration in the X-axis, gateway acceleration in the Y-axis, gateway acceleration in the Z-axis, gateway battery level measured in millivolts (mV), gateway radio channel, gateway transmission power measured in decibels milliwatt (dBM), gateway antenna orientation, clientʼs (senderʼs) media access control (MAC) address, clientʼs channel check rate, clients Temperature (mC), clientʼs received signal strength indicator (RSSI) measured in decibels milliwatt (dBM), the Link Quality Indicator (LQI), the variables of interest are the client's acceleration in the X-axis, Y-axis, and Z-axis, the client's battery level measured in millivolts (mV), the client's radio channel, the client's transmission power measured in decibels-milliwatt (dBM), and the client's antenna orientation.

### 4.3 Scenarios

The experiment was conducted in the IoT & Wireless Communication Protocols Lab at the ECE/KoE/IIUM. The location was chosen to be free from obstructions and wireless activity to minimize any potential external sources of interference and disturbance that could affect the accuracy of the results. The transmitter transmits a single packet while the receiver effectively acquires and archives the RSSI (Received Signal Strength Indicator), LQI (Link Quality Indicator), and other relevant metrics associated with each packet received from the sender. The clients, referred to as Senders, are positioned at distances of 1 m, 2 m, and 3 m from the Gateway, which acts as the receiver. The data were collected during 24-hour time intervals for individual nodes. This was done to investigate the impact of day and night temperature on the other parameters, such as RSSI and LQI.

**Fig. 1:**
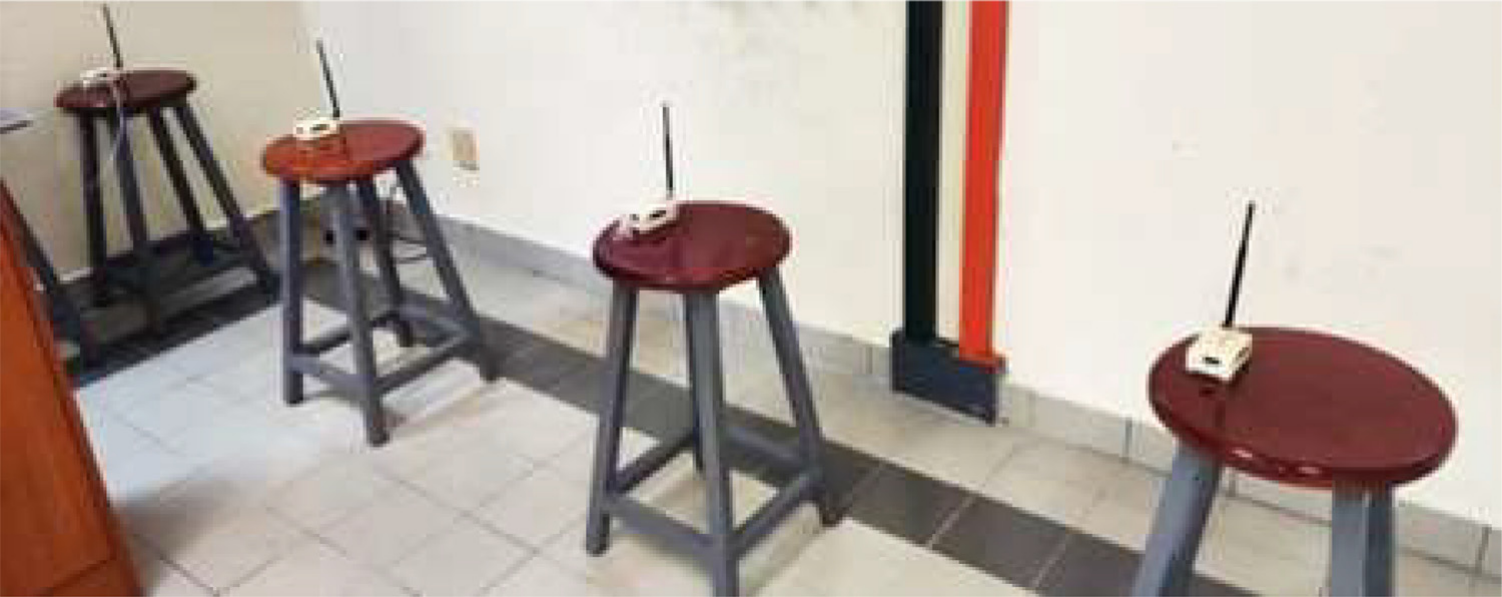
Lab Setup for Data Collection (Horizontal).

**Fig. 2:**
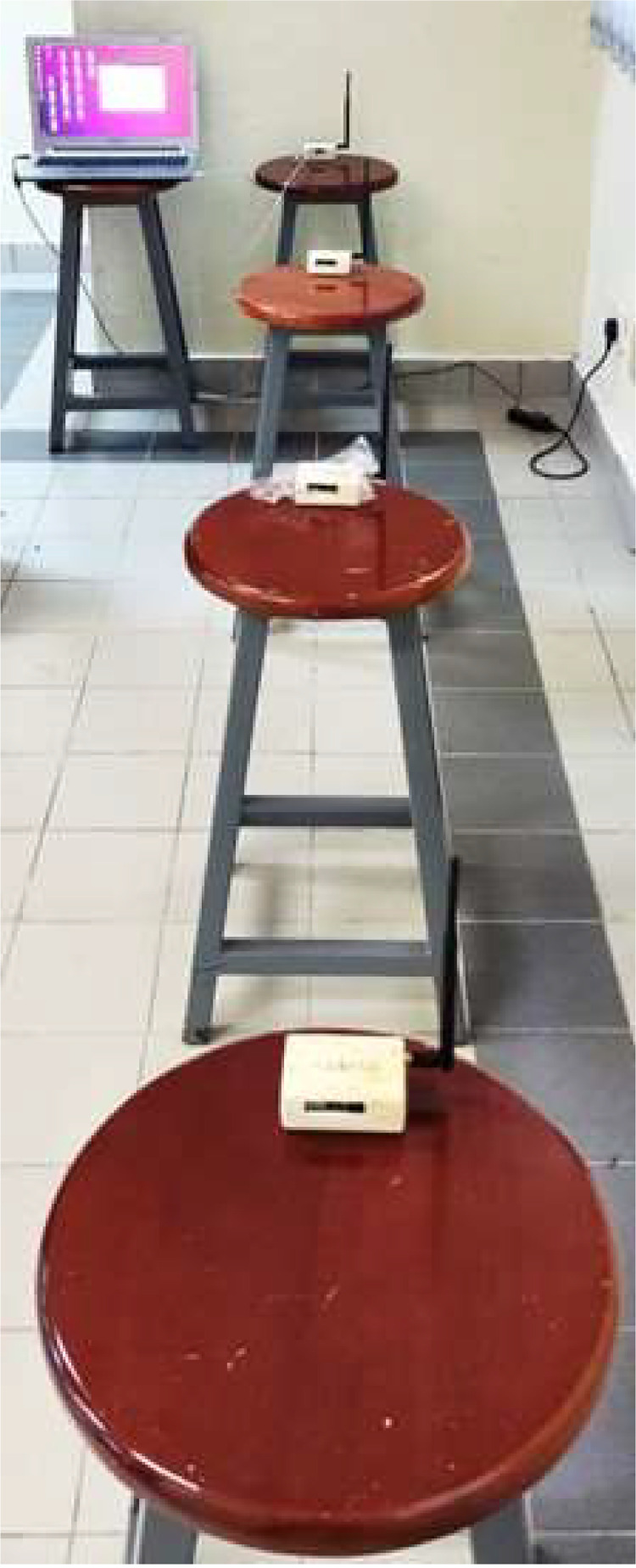
Lab Setup for Data Collection (Vertical).

Furthermore, we alter the orientation of the antennas and collect the corresponding data. To provide a visual representation, we assigned the client nodes as X1, X2, and X3, with X1 positioned at a distance of 1 m, X2 set at a distance of 2 m, and X3 positioned at a distance of 3 m from the Gateway, as depicted in Fig. 1, Fig. 2.

A total of 347,200 instances were gathered, with X1 instances occurring at a distance of 1 m, totalling 107,396, X2 instances occurring at a distance of 2 m, totalling 126,607, and X3 instances occurring at a distance of 3 m, totalling 113,197.

Out of the total data collected, 85,984 instances were gathered for X1 when the antenna orientation was 90°, 10,562 instances were collected for X1 when the antenna orientation was set at 180°, and 10,850 instances were collected for X1 when the antenna orientation was set at 0°. Similarly, instances 84,016, 3849, and 38,742 of X2 were gathered with corresponding Antenna Orientations of 90°, 180°, and 0°, respectively. Similarly, 98,651 instances were gathered for X3 when the antenna orientation was 90°, 7037 instances were collected when the antenna orientation was set at 180°, and 7509 instances were collected when the antenna orientation was set at 0°.

**Fig. 3:**
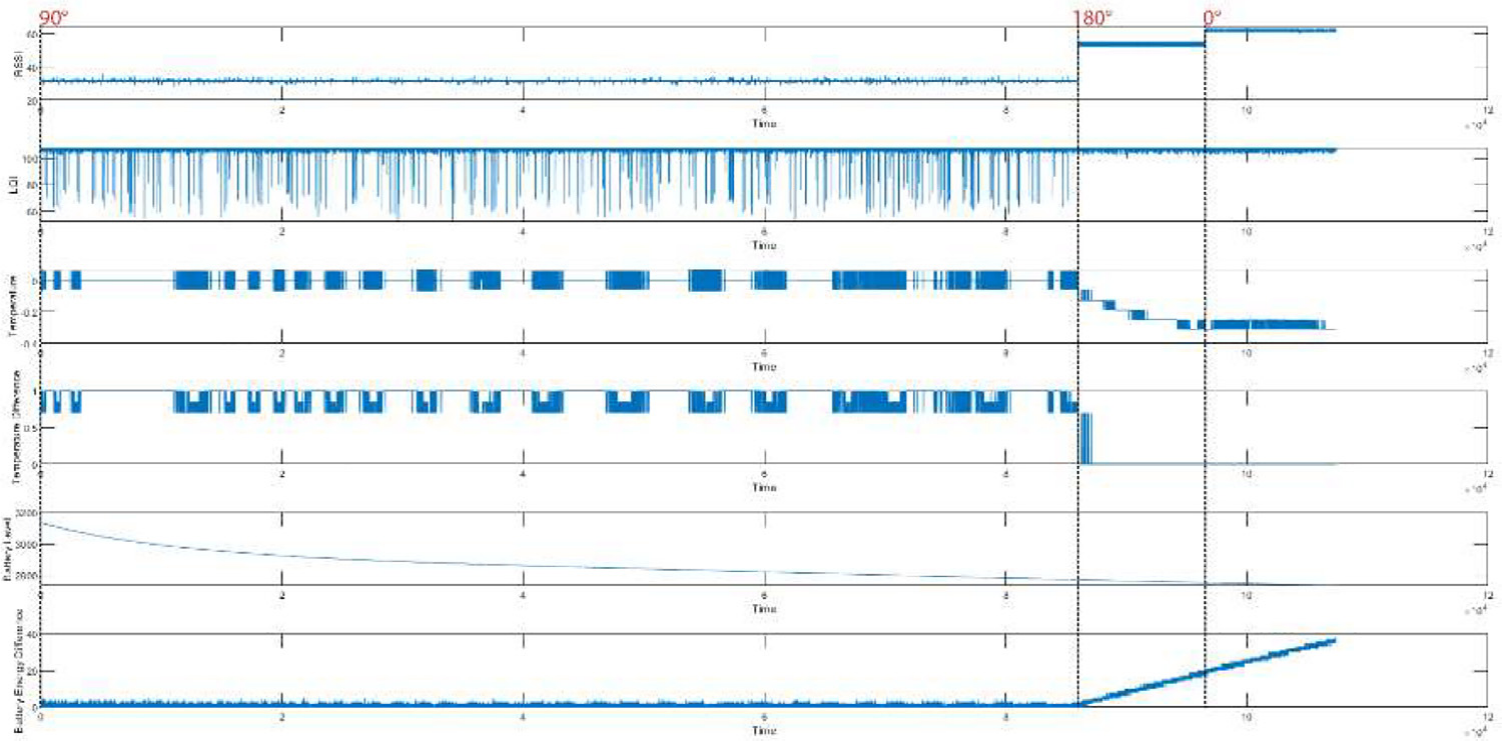
Data Representation of Client-X1.

**Fig. 4:**
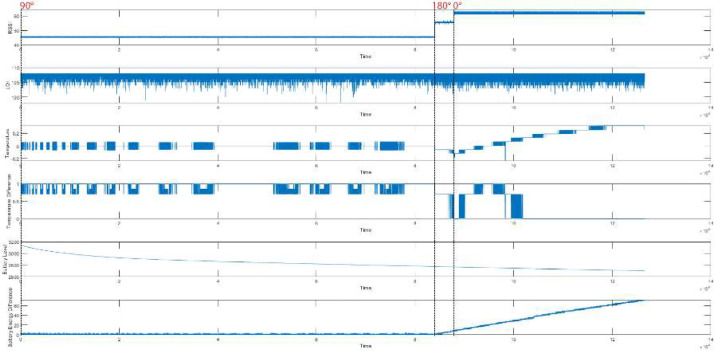
Data Representation of Client-X2.

**Fig. 5:**
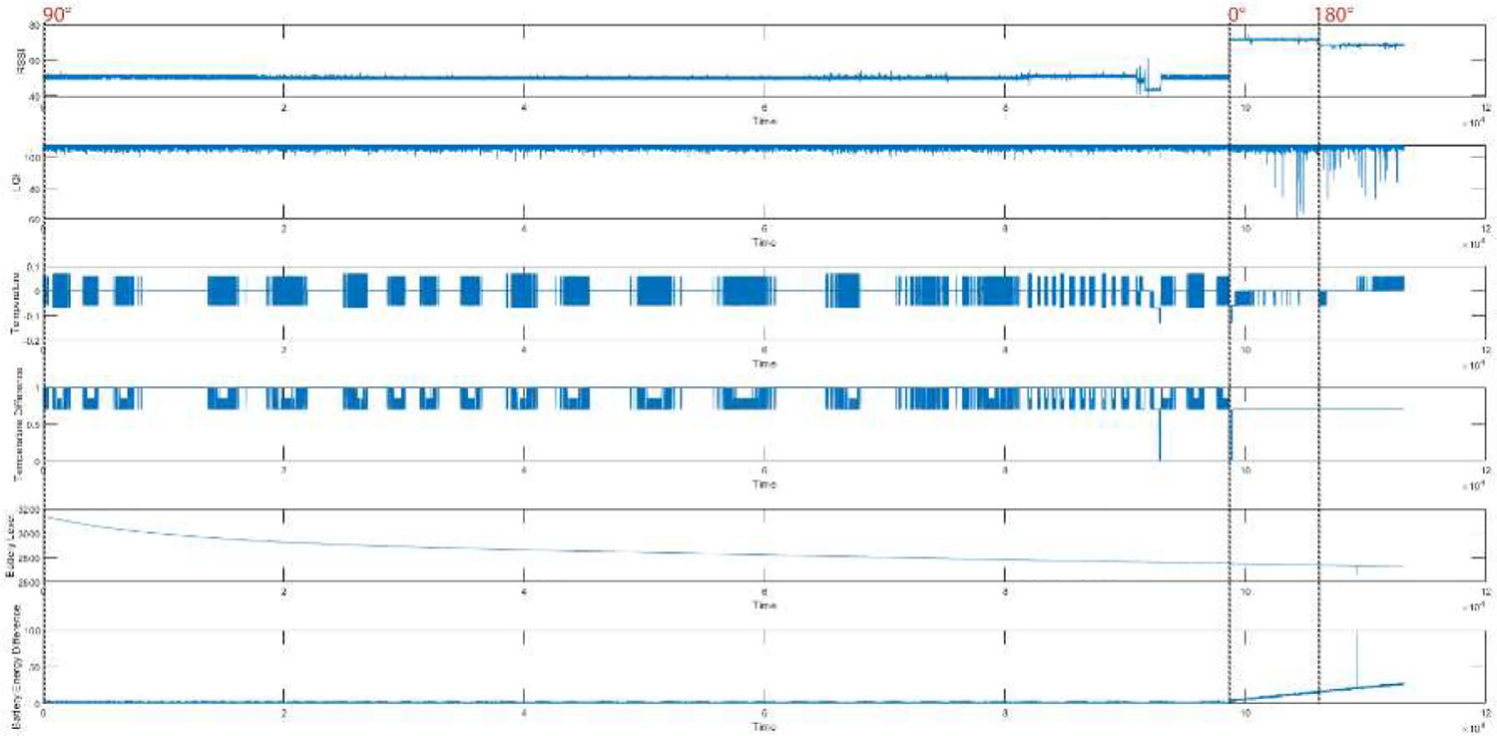
Data Representation of Client-X3.

Likewise, we considered the clients with 90-degree antenna orientation are not malicious, and by altering the MAC of the clients, it can be possible to treat X2′s and X3′s requests as malicious requests for client X1 and vice versa. We represent the Temperature and Battery Level (mV) difference along with other metrics for client-X1 in Fig. 3, client-X2 in Fig. 4, and client-X3 in Fig. 5. Moreover, statistical measurements at different angles were also performed, as depicted in Table 2, Table 3, Table 4. It was observed that a slight shift in distance and antenna orientation directly impacts the device's RSSI value. Moreover, the LQI value is also affected by environmental nature changes such as airflow change, day or night time, environmental temperature, noise, and vibration of the lab building.

**Table 2:** Statistical Measurements of end node at a 1-meter distance from the Gateway.

Antenna Orientation	90°		0°		180°	
	RSSI	LQI	RSSI	LQI	RSSI	LQI
Mean	−31.27109695	107.0796544	−61.43105991	107.06553	−52.99857981	107.2227798
Median	−31	107	−61	107	−53	107
Mode	−31	107	−61	107	−53	108
StandardDeviation	0.469691642	2.319720628	0.582167548	0.919088555	0.662191909	0.868430152
Variance	0.220610238	5.381103792	0.338919053	0.844723772	0.438498125	0.754170929
Skewness	−1.105996839	−15.45736309	−0.861025895	−1.14570095	−1.506213501	−1.451732169
Kurtosis	4.631642131	283.9018522	2.963930892	4.937040639	6.838363247	6.757440566
Peak2Peak	8	56	4	7	3	8
RMS	31.27462409	107.1047778	61.43381812	107.0694744	53.00271615	107.2262962
CrestFactor	−0.895294534	1.008358378	−0.976660768	1.008690858	−0.981081797	1.007215616
ShapeFactor	1.000112792	1.000234623	1.000044899	1.000036842	1.000078046	1.000032796
ImpulseFactor	−0.895395516	1.008594963	−0.97670462	1.00872802	−0.981158367	1.007248648
MarginFactor	−0.028633326	0.009419109	−0.015899199	0.009421595	−0.018512918	0.00939398
Energy	84,101,132	986,359,731	40,949,137	124,383,015	29,671,699	121,436,369

**Table 3:** Statistical Measurements of end node at 2-meter distance from the Gateway.

Antenna Orientation	90°		0°		180°	
	RSSI	LQI	RSSI	LQI	RSSI	LQI
Mean	−45.34716006	107.4471767	−61.38544732	107.0470291	−55.04416732	107.0579371
Median	−45	108	−61	107	−55	107
Mode	−45	108	−61	107	−55	107
StandardDeviation	0.476095008	0.740152437	0.581259055	0.910749826	0.223659995	0.928314148
Variance	0.226666457	0.54782563	0.337862089	0.829465245	0.050023793	0.861767157
Skewness	−0.641763094	−1.793927314	−1.225135728	−1.11254814	−4.464017589	−1.271009306
Kurtosis	1.412786458	9.052677157	3.50121769	4.968573042	26.98580474	5.730873384
Peak2Peak	2	10	4	7	3	7
RMS	45.3496592	107.4497259	61.38819916	107.0509032	55.04462159	107.0619608
CrestFactor	−0.970238824	1.005121223	−0.977386547	1.008865846	−0.981022277	1.008761648
ShapeFactor	1.000055111	1.000023725	1.000044829	1.000036191	1.000008253	1.000037584
ImpulseFactor	−0.970292295	1.00514507	−0.977430362	1.008902358	−0.981030373	1.008799561
MarginFactor	−0.02139698	0.009354783	−0.015922835	0.009424852	−0.017822604	0.009422931
Energy	172,786,599	970,001,990	145,999,653	443,979,286	11,662,125	44,118,252

**Table 4:** Statistical Measurements of end node at 3-meter distance from the Gateway.

Antenna Orientation	90°		0°		180°	
	RSSI	LQI	RSSI	LQI	RSSI	LQI
Mean	−50.36807534	107.1540278	−71.78053003	107.039153	−68.08426886	107.2327696
Median	−51	107	−72	107	−68	107
Mode	−51	108	−72	107	−68	108
StandardDeviation	1.133196352	0.910570615	0.429082835	1.535431692	0.363372133	1.46567333
Variance	1.284133973	0.829138845	0.184112079	2.357550481	0.132039307	2.148198311
Skewness	2.67493682	−1.329461494	1.353074851	−14.5055322	−0.426368052	−11.60009444
Kurtosis	15.68617943	6.068829208	4.804539184	372.1739341	108.9152468	209.3796097
Peak2Peak	22	11	6	47	16	35
RMS	50.38082109	107.1578966	71.78181231	107.0501635	68.0852384	107.2427843
CrestFactor	−0.774104097	1.007858528	−0.961246279	1.008872816	−0.895935763	1.007060762
ShapeFactor	1.000253052	1.000036105	1.000017864	1.000102864	1.00001424	1.000093391
ImpulseFactor	−0.774299985	1.007894917	−0.961263451	1.008976594	−0.895948521	1.007154812
MarginFactor	−0.015372832	0.009406039	−0.013391702	0.009426239	−0.013159406	0.00939223
Energy	250,398,645	1,132,791,164	38,691,088	86,051,169	32,620,715	80,932,641

### 4.4 Potential real-world applications

Our exploration of the practical deployment scenarios for the novel dataset on authentication and authorization using physical layer properties in indoor environments spans a range of applications that are particularly useful for training machine learning models in various IoT applications. Here, we elucidate how these scenarios can be implemented in real-world applications:

*Secure IoT Deployments in Smart Buildings:* The detailed examination of RSSI, LQI, and other metrics in different antenna orientations and distances provides invaluable insights for enhancing security protocols in smart buildings. Implementing our findings can optimize device authentication and maintain integrity in densely networked environments like modern commercial complexes and residential areas. By leveraging the unique physical layer properties captured in our dataset to train machine learning models, architects can design more robust security frameworks that are resilient against common attack vectors such as signal spoofing and interference.

*Industrial Automation and Control Systems:* In industrial settings, the reliability and security of sensor and communication networks are paramount. Our research outcomes, focusing on the stability of communication channels in varying environmental conditions, offer critical data for developing advanced authentication systems and training machine learning models. These systems can effectively differentiate between genuine and rogue devices, thus safeguarding critical industrial processes from unauthorized access and ensuring continuous operation without disruption.

*Healthcare Monitoring Systems:* In healthcare facilities, where the deployment of IoT devices is rapidly expanding to include patient monitoring systems, ensuring data privacy and security is critical. The authentication techniques refined through our study can be directly applied to verify devices within these networks, enhancing patient data confidentiality and preventing unauthorized access to sensitive health information.

*Retail and Inventory Management:* The ability to authenticate and authorize devices reliably in a dynamic environment like retail stores, where IoT devices are increasingly used for inventory tracking and customer service, can benefit immensely from our findings. The physical layer data characteristics derived from our dataset can help accurately identify and authorize devices, thus preventing inventory shrinkage and ensuring accurate data collection for inventory and sales purposes.

*Smart Home Ecosystems:* Our dataset's practical application extends to smart homes, where multiple IoT devices must interact securely. Utilizing the physical layer properties for authentication ensures that only legitimate devices can communicate within the network, thus enhancing the overall security of the smart home ecosystems against potential cyber threats.

## Limitations

The dataset is not suitable for scenarios involving nodes with mobility.

## Ethics Statement

The authors have adhered to the ethical guidelines for publication in Data in Brief and have verified that the present study does not involve human participants, animal experimentation, or data obtained from social media platforms.

## CRediT Author Statement

**Kazi Istiaque Ahmed:** Conceptualization; Data curation; Formal analysis; Investigation; Methodology; Roles/Writing - original draft; Writing - review & editing. **Mohammad Tahir**: Conceptualization; Methodology; Validation; Analysis; Investigation; Roles/Writing - original draft; Writing - review & editing. **Sian Lun Lau**: Conceptualization; Validation; Roles/Writing - original draft; Writing - review & editing. **Mohamed Hadi Habaebi:** Conceptualization; Methodology; Validation; Roles/Writing - original draft; Writing - review & editing. **Abdul Ahad:** Validation; Roles/Writing - original draft; Writing - review & editing. **Ivan Miguel Pires:** Funding acquisition; Validation; Roles/Writing - original draft; Writing - review & editing.

## Data Availability

Dataset for Authentication and Authorization using Physical Layer Properties in Indoor Environment (Original data) (Zenodo).
